# Interface Engineering Suppresses Self‐Annealing in Electroplated Nanograined Copper for Low‐Temperature Copper‐to‐Copper Bonding

**DOI:** 10.1002/advs.202521964

**Published:** 2026-01-04

**Authors:** Gangqiang Peng, Xinyi Dong, Binzhao Li, Chuan He, Cong Chen, Licheng Huang, Jian Liu, Yonghan Zhou, Qikai Li, Hao Gong, Yuechen Wang, Kaiyu Mu, Sheng‐Jye Cherng, Po‐Yen Yang, Yang Lu, Yong Yang, Shih‐Wei Hung, Shien‐Ping Feng

**Affiliations:** ^1^ Department of Systems Engineering City University of Hong Kong Kowloon Hong Kong China; ^2^ Department of Physics City University of Hong Kong Kowloon Hong Kong China; ^3^ Department of Mechanical Engineering City University of Hong Kong Kowloon Hong Kong China; ^4^ Hong Kong Science and Technology Parks ASM Pacific Technology Ltd. Hong Kong China; ^5^ Nano and Advanced Materials Institute Hong Kong Science and Technology Parks Hong Kong China; ^6^ Doctech. HK Ltd. Hong Kong Science and Technology Parks Hong Kong China; ^7^ Department of Mechanical Engineering The University of Hong Kong Pokfulam Hong Kong China; ^8^ Department of Materials Science and Engineering City University of Hong Kong Kowloon Hong Kong China

**Keywords:** advanced electronic packaging, Cu─Cu direct bonding, low‐temperature bonding, nanograined Cu

## Abstract

Nanograined copper (ng‐Cu) attracts interest in advanced electronic packaging due to its grain boundary–mediated fast diffusion, enabling low‐temperature copper‐to‐copper (Cu‐Cu) bonding. However, after electroplating ng‐Cu, self‐grain growth at room temperature (self‐annealing) during prolonged processing time limits its practical use in manufacturing. This work proposes an ng‐Cu layer atop a (111)‐oriented nanotwinned Cu substrate (ng/nt‐Cu) that resists self‐annealing. The Cu(111) interface between the ng and nt layers resists grain boundary migration due to uniform atomic energy, which inhibits atom adsorption. Moreover, electroplating on the nt‐Cu substrate promotes the formation of a high density of Σ3 twin boundaries and stacking faults in the ng layer, relaxing internal stress and suppressing grain boundary motion. As a result, the ng/nt‐Cu retains its microstructure for over 30 days and enables high‐quality Cu‐Cu bonding at a low thermal budget. This study provides crystallographic insights of electroplated copper and advances the low‐temperature Cu─Cu bonding technique in electronic packaging.

## Graphical abstract

A (111)‐oriented nanotwinned copper substrate stabilizes the deposited nanograined copper and prevents self‐annealing by restricting grain boundary motion at the interface and promoting the formation of stacking faults within the nanograined copper. Its preserved nanostructure maintains bonding capability even after 30 days of storage, resulting in high‐quality low‐temperature Cu‐Cu bonding.

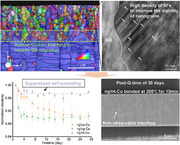

## 1 Introduction

For decades, increased computational power has been largely propelled by greater transistor density [1, 2, 3]. However, as continued transistor scaling approaches fundamental physical limits, alternative strategies to enhance chip performance are emerging [4, 5, 6, 7]. Advanced packaging technology, which stacks IC chips vertically to increase transistor density, offers a promising approach to extending Moore's Law, with additional benefits of shortening electrical pathways, reducing power consumption, and accelerating design‐to‐market cycles [8, 9, 10].

When increasing I/O density by reducing pitch below 10 µm, soldering and C2 bonding become unfeasible due to liquid bridging risks and the formation of brittle, high‐resistance intermetallic compounds (IMCs), raising reliability concerns [11, 12, 13, 14]. To overcome these challenges, an alternative approach is to replace solder with direct Cu─to─Cu (Cu─Cu) bonding, allowing for further reduction in pitch [9, 15]. The two main techniques for direct Cu─Cu bonding are thermocompression bonding (TCB), which applies simultaneous pressure and heat, and hybrid bonding (HB), which bonds both copper surfaces and the surrounding dielectric [16].

Currently, for standard coarse‐grained Cu (cg‐Cu), successful Cu bonding typically requires temperatures above 300°C for hours [17, 18]. The temperature exceeds the melting points of traditional eutectic SnPb and SnAgCu solders, with reflow typically below 250°C in minutes [19, 20]. High bonding temperatures and prolonged processing time pose industrial challenges, risking device damage and limiting the integration of temperature‐sensitive chips for future heterogeneous 3DIC stacking. Additionally, elevated temperatures increase residual stresses and cause misalignment, raising concerns about production yields [21].

To reduce the thermal budget of Cu─Cu direct bonding, researchers have explored methods such as plasma surface activation [22], passivation layers (e.g., Ag, Au) [23], oxidation‐resistant organic monolayers [24], and Cu grain engineering, including (111)‐oriented nanotwinned Cu (nt‐Cu), (110)‐oriented nanotwinned Cu (p‐nt‐Cu), and nanograined Cu (ng‐Cu) [25, 26, 27, 28, 29]. Among these, grain engineering stands out, requiring only electroplating additives and adjustments to refine the microstructure, and avoiding costly additional processing. Notably, ng‐Cu, with its abundant grain boundaries (GBs) serving as diffusion pathways, enables cross‐interface bonding that enhances quality and reliability. Note that solid‐state bonding is a diffusion‐controlled metallic creep process dominated by Coble creep at moderate temperatures, with kinetics accelerated by reducing the grain size [30, 31].

Despite its advantages, electroplated ng‐Cu undergoes spontaneous grain growth at room temperature (self‐annealing), degrading its microstructure within days [32]. This instability poses a serious barrier to industrial adoption, as wafers are typically electroplated in the fab and then shipped to outsourced assembly and test (OSAT) companies; this queue‐time (Q‐time) for transport and storage may exceed one week, long enough for ng‐Cu to lose bonding activity [33]. Thinning the ng‐Cu layer can reduce residual stress and slow self‐annealing [34]. A thin ng‐Cu layer atop coarse‐grained copper (ng/cg‐Cu) is a potential solution [27, 35]. However, in this study, we found that this structure experiences unexpected curvature‐driven grain boundary migration at the ng/cg interface, rapidly consuming the nanograined layer despite its reduced thickness, with this abnormal, interface‐driven grain growth occurring even faster than natural self‐annealing. Therefore, strategies are needed to stabilize ng‐Cu over extended Q‐time, enabling its practical integration in semiconductor packaging.

In this work, a bilayer structure was fabricated by electroplating ng‐Cu on top of nt‐Cu, forming ng/nt‐Cu. This design effectively suppresses self‐annealing and preserves the nanograined microstructure during extended room‐temperature storage for over 30 days. The stability arises from two key mechanisms. First, the ng/nt interface resists GB migration owing to the atomically flat, low‐step‐density Cu(111) facet, which minimizes dangling bonds and adatom adsorption, thereby reducing out‐of‐plane diffusivity. Second, during ng‐Cu electroplating on nt‐Cu, the twin boundaries of the nt substrate template the formation of Σ3 twin boundaries in the ng‐Cu. A higher density of Σ3 twins within nanograins further enhances stability by strongly suppressing GB mobility at triple junctions. As a result, reliable low‐temperature Cu─Cu bonding can be achieved even after one month of storage, producing bonding joints of high quality, superior mechanical strength, and excellent resistance to electromigration.

## 2 Results and Discussion

### 2.1 Characterization of the ng/nt‐Cu and its Suppressed Self‐Annealing

**FIGURE 1:**
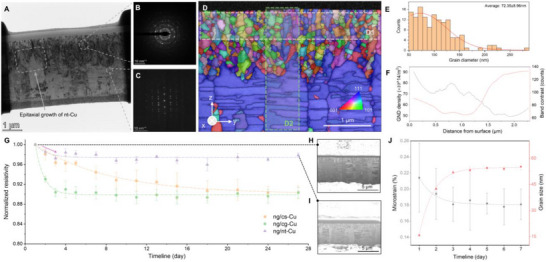
Characterization of the ng/nt‐Cu bilayer and post‐electroplating microstructural evolution. (A) Cross‐sectional bright‐field TEM image revealing the heterogeneous microstructure. (B) SAED of the ng‐Cu layer, confirming fine, randomly oriented nanograins. (C) SAED of the nt‐Cu layer, showing twins with Σ3{111} boundaries normal to the film plane. (D) Cross‐sectional TKD inverse pole Figure (IPF‐z) map; colors indicate crystallographic orientations (see stereographic triangle, bottom right). (E) Grain size distribution from region D1 in (D). (F) Geometrically necessary dislocation (GND) density and band contrast profile across region D2 (bottom to surface). (G) Post‐electroplating resistivity evolution of atop ng‐Cu in ng/cs‐Cu, ng/cg‐Cu, and ng/nt‐Cu. The ng‐Cu layer thickness in each group is ∼1.76 µm. (H, I) Cross‐sectional FIB images of ng/nt‐Cu at day 1 and day 27, respectively. (J) Post‐synthesis microstrain and grain size evolution of atop ng‐Cu in ng/nt‐Cu, derived from W‐H analysis of χ = 45°‐tilted XRD data.

Microstructural characterization of ng/nt‐Cu was performed using scanning transmission electron microscopy (STEM) and transmission Kikuchi diffraction (TKD). Characterizations were performed five days after electroplating, when the ng/nt‐Cu microstructure had stabilized. The ng‐Cu layer consisted of fine, randomly oriented grains, while the underlying nt‐Cu exhibited columnar twin structures with out‐of‐plane {111} twin planes, confirmed by selected‐area electron diffraction (SAED, Figure 1A–C). Note that the zig‐zag interface between ng‐Cu and nt‐Cu, arising from the epitaxial growth of additional nt‐Cu during the initial electroplating of ng‐Cu on a flat nt‐Cu substrate, as illustrated in Figure S1. Higher‐magnification TEM (Figure S2) revealed the presence of defects, including dislocations and stacking faults, in ng‐Cu; the nt‐Cu underlayer consisted of large grains and contained intragranular nanoprecipitates.

In Figure 1D, TKD mapping of region D1 (ng‐Cu) shows an average grain size of 72.35 nm (Figure 1E). Analysis of region D2 further reveals that the geometrically necessary dislocation (GND) density increases from the substrate toward the ng/nt‐Cu surface, with ng‐Cu consistently exhibiting higher GND density than nt‐Cu due to intrinsic structural differences (Figure 1F and Figure S3). GND density increased with film thickness, reflecting accumulated internal electroplating stresses and explaining the preferential initiation of self‐annealing at the surface [36]. Additionally, the inverse pole figure revealed a weak <322> texture along the film‐normal (z) direction in the ng‐Cu layer (Figure S4), which is believed to contribute negligibly to stability.

We investigated the self‐annealing behavior of ng‐Cu films with identical thickness, deposited on three types of substrates: (111)‐oriented Cu seed (cs‐Cu), cg‐Cu, and nt‐Cu. Here, the roughness of the three substrates (cs‐Cu, cg‐Cu, nt‐Cu) was standardized and treated with electropolishing prior to electroplating (Figure S7). Impurity concentrations were measured by time‐of‐flight secondary ion mass spectrometry (ToF‐SIMS, Figure S8) for ng‐Cu, nt‐Cu, and cg‐Cu. The ng‐Cu contained ∼283 ppm of impurities (C, S, Cl), whereas both nt‐Cu and cg‐Cu exhibited lower but comparable levels (30–60 ppm). These similar impurity concentrations indicate that impurities do not influence the self‐annealing behavior of the atop ng‐Cu by the Gorsky effect [37, 38].

To evaluate self‐annealing, the resistance of the atop ng‐Cu layers was measured using four‐probe techniques and calculated with an equivalent circuit model (Figure S9), combined with focused ion beam (FIB) analysis (Figure 1G–I). In the ng/cs‐Cu, self‐annealing progressed steadily over one month, initiating at the top surface (Figure S10), due to internal stress relaxation and surface energy minimization [36]. In the ng/cg‐Cu, grain growth was much faster, with resistance decreasing to approximately 90% within three days. FIB imaging (Figure S11) revealed that the ng/cg interface drives rapid bottom‐to‐top GB migration, with the cg‐Cu coalescing and consuming the ng‐Cu layer. The ng/nt‐Cu demonstrated exceptional stability (>49 days), with only minor resistance reduction observed in the first two days (Figure 1G–I, Figure S12). Furthermore, resistance measurements of ng‐Cu films with varying thicknesses on different substrates (Figure S13) showed that thicker layers self‐anneal more rapidly due to higher accumulated internal stress, whereas ng/nt‐Cu consistently exhibited superior stability across all thicknesses.

To probe the microstructural evolution in ng/nt‐Cu, χ = 45°‐tilted X‐ray diffraction (XRD) was performed over seven days, ensuring that all diffraction data originated from the ng‐Cu layer (see Supporting Information). Williamson‐Hall (W–H) analysis was used to deconvolute nanograin size and microstrain from the full‐width‐half‐maximum (FWHM) values [39, 40]. As shown in Figure 1J, XRD revealed rapid coarsening of ng/nt‐Cu nanograins during the first two days after electroplating, followed by stabilization. Microstrain showed a similar trend, decreasing sharply before reaching a plateau. The early‐stage coarsening reflects relaxation of high initial defect densities, after which the driving force diminishes as grains enlarge. The mechanisms responsible for subsequent stabilization are discussed below.

### 2.2 Cu(111) Interface Resists GB Migration

**FIGURE 2:**
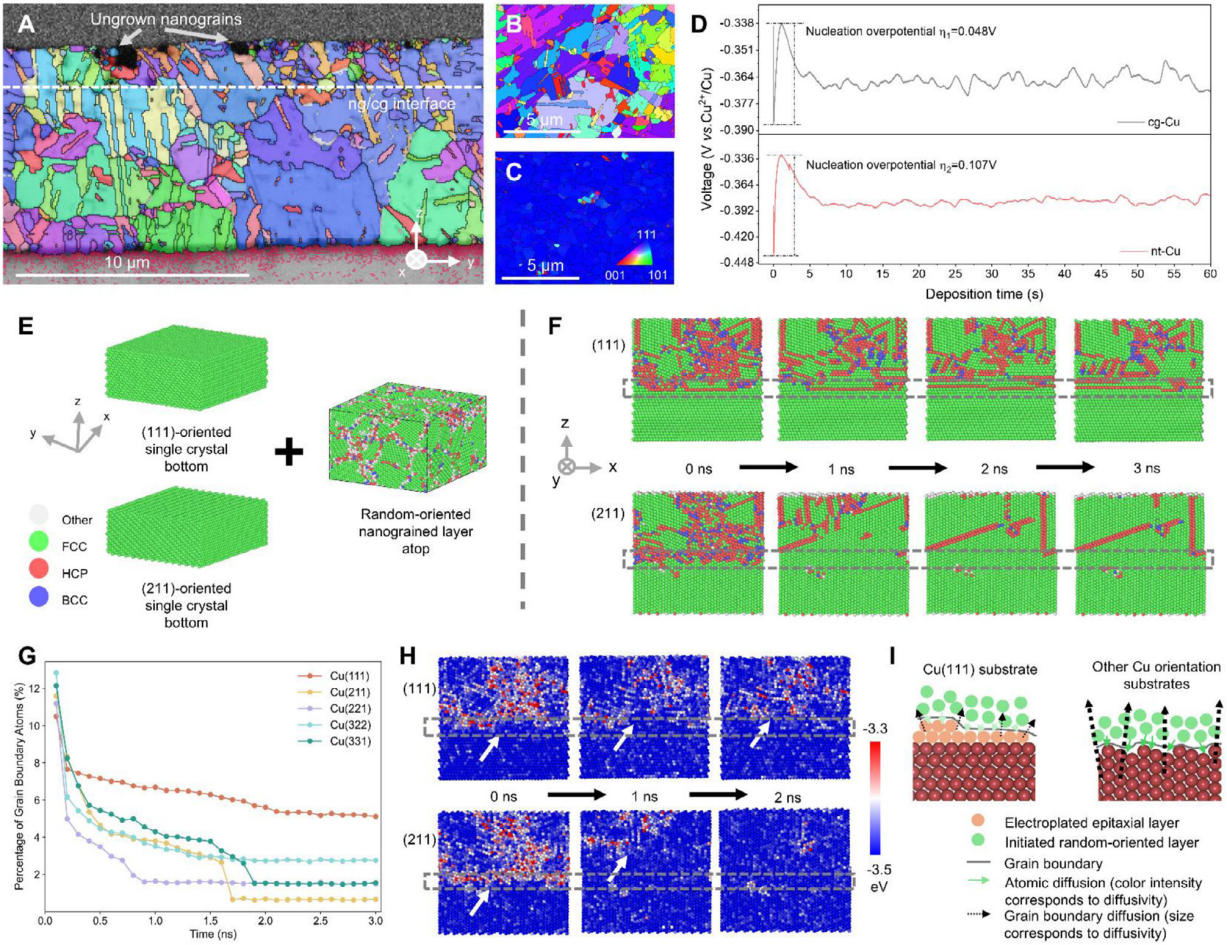
Comparative interface mechanisms of Cu(111) and other Cu facets. (A) Cross‐sectional EBSD IPF‐z map of ng/cg‐Cu after 3 days. Gray arrows and dotted line indicate ungrown nanograins (limited by EBSD resolution) and the ng/cg‐Cu interface, respectively. (B, C) Surface EBSD IPF‐z maps of cg‐Cu and nt‐Cu substrates, respectively. Color key references the stereographic triangle. (D) Galvanostatic electroplating profiles for ng‐Cu on both substrates at 6 ASD. (E) Molecular dynamics models: randomly oriented ng‐Cu atop (i) (111)‐oriented or (ii) (211)‐oriented single‐crystal substrates. (F) Simulated isothermal annealing; dotted frames mark initial interface positions. The (111) interface remains coherent after 3 ns, while the (211) interface dissolves. (G) Temporal evolution of GB atom percentage, showing maximal retention for (111) systems. (H) Atomic energy evolution during annealing; dotted frames indicate initial interface positions and arrows track interfacial displacement. (I) Schematic illustration of atomic diffusion and GB migration with substrates of Cu(111) and other Cu facets.

Although both ng/nt‐Cu and ng/cg‐Cu are double‐layer designs, their differing stability highlights fundamentally distinct underlying mechanisms. Cross‐sectional FIB (Figure S11) and EBSD mapping of the ng/cg‐Cu interface after three days (Figure 2A) revealed rapid consumption of nanograins by adjacent coarse grains, occurring even faster than self‐annealing in the ng‐Cu layer. Interestingly, this phenomenon is absent in ng/nt‐Cu (Figure S12).

Considering the possible role of impurities in the ng/nt (or ng/cg) interface region via Zener pinning, depth‐profiling ToF‐SIMS (Figure S14) was used to assess impurity distributions [41]. Both systems showed comparable impurity levels at the interface. While ng/cg‐Cu displayed a steeper impurity gradient across the interface, local average impurity concentrations were similar or even higher than those in ng/nt‐Cu, ruling out impurity‐driven stabilization as the primary stabilization mechanism in their interface region. Notably, after 30 days of room‐temperature storage, C, H, O, and S impurities in ng/nt‐Cu redistributed toward the free surface throughout the film, while Cl migration was confined to the top ng‐Cu layer. This redistribution is attributed to stress‐induced diffusion (Gorsky effect) [42, 43], and the distinct behavior of Cl versus other impurities likely stems from stronger Cu–Cl binding (see Supporting Information for details). Such redistribution was absent in ng/cg‐Cu, where rapid self‐annealing occurred post‐electroplating, confirming that GBs serve as primary diffusion pathways [44]. Similarly, annealing under 200°C did not induce long‐range impurity transport in either system, as grain growth eliminated GB networks.

According to classical grain growth theory, spontaneous coarsening is curvature‐driven: the greater the curvature difference, the stronger the driving force [45, 46]. This explains the rapid nanograin consumption observed at the ng/cg‐Cu interface. However, despite a similar curvature mismatch at the ng/nt‐Cu interface, no curvature‐driven grain growth occurred, indicating that interfacial crystallographic orientation plays an important role. EBSD analysis confirmed distinct substrate facets: cg‐Cu exhibited random orientations, whereas nt‐Cu showed uniformly (111)‐oriented (Figure 2B, C). At the microscopic level, grain growth involves atom desorption from high‐curvature grains and adsorption across the GB onto low‐curvature grains. Because atomic configurations differ among Cu facets (Figure S18), desorption–adsorption kinetics also vary. Electroplating profiles (Figure 2D) revealed that the nucleation overpotential for ng‐Cu on nt‐Cu is nearly twice that on cg‐Cu, indicating that Cu ions adsorb less readily on (111) surfaces. Previous studies likewise report reduced oxygen adsorption and enhanced oxidation resistance on Cu(111) [47]. Together, these results support our conclusion that the (111) facet stabilizes the ng/nt‐Cu interface.

To further investigate the facet effect in an atomic perspective, molecular dynamics (MD) simulations were performed, placing a randomly oriented ng‐Cu layer atop single‐crystal Cu layers of different orientations (Figure 2E, Figure S19). After 3 ns of isothermal equilibration at 300 K, most orientations showed interface elimination via upward grain growth, except for Cu(111), which retained a well‐defined interface (Figure 2F, Figure S20). Traditional MD analysis may misidentify low‐energy boundaries such as Σ3 twins as grain boundaries, and thus incorrectly track interface evolution. To resolve this, we defined the interface region by atomic potential energy, identifying atoms with energies above −3.4 eV as GB atoms (Figure S21). Statistical analysis showed that Cu(111) maintained the highest fraction of GB atoms, supporting its interface stability (Figure 2G). Further, atomic energy analysis revealed that the Cu(111) interface migrates only slightly upward, indicating slow kinetics, while interfaces with other orientations move more rapidly (Figure 2H, Figure S20). Mean square displacement (MSD) analysis along the interface normal (z‐direction) confirmed that Cu atoms in Cu(111) exhibit the lowest mobility (Figure S22). Collectively, these results indicate that the Cu(111) interface has the lowest atomic and grain boundary diffusivity (schematic, Figure 2I).

This kinetic stability is rooted in differences in surface energy among Cu facets (Figure S23). High‐index facets, with abundant kink and step sites, possess higher surface energy and more undercoordinated atoms than the close‐packed Cu(111) surface, leading to increased instability and atomic mobility [48]. This facilitates faster grain boundary migration and interface kinetics at high‐index facets. In contrast, the close‐packed Cu(111) facet offers greater atomic coordination and reduced mobility, leading to enhanced interfacial stability.

### 2.3 Templated ∑3 Twin Boundary for Enhanced Stability

**FIGURE 3:**
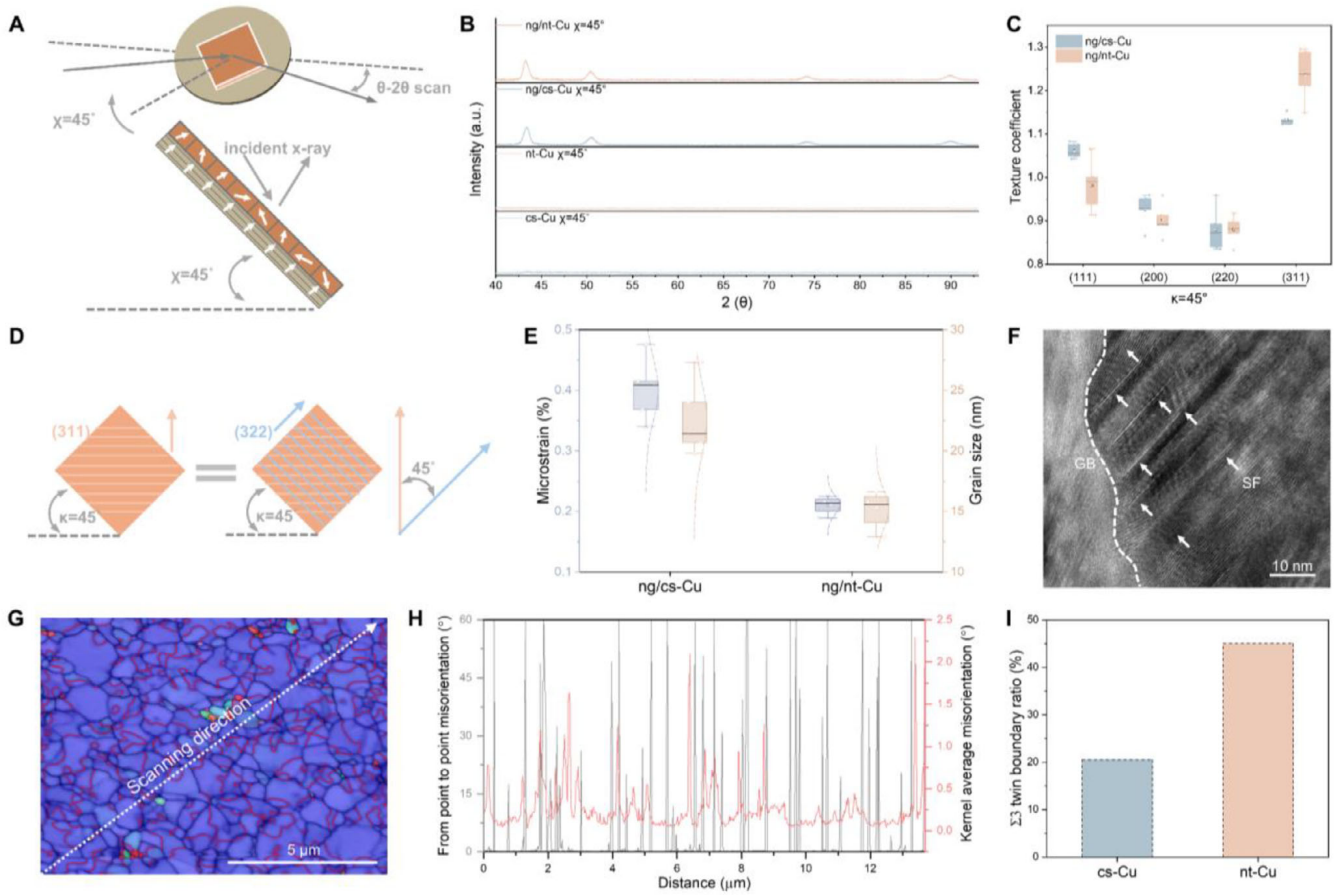
Structural analysis of the atop ng‐Cu layer on nt‐Cu. (A) Schematic of the χ  =  45°‐tilted XRD measurement. The lower light brown region represents the (111)‐oriented substrates (nt‐Cu and cs‐Cu), with parallel lines showing (111) planes and white arrows indicating their normals. The upper orange layer depicts the randomly oriented ng‐Cu layer, with disordered white arrows representing the random grain orientations. (B) χ  =  45°‐tilted XRD patterns of bare nt‐Cu and cs‐Cu substrates and of the ng‐Cu layers deposited atop them, demonstrating effective suppression of substrate signals. (C) Texture coefficients of ng‐Cu layers on nt‐Cu and cs‐Cu substrates. (D) Schematic illustrating the relationship between χ  =  45°‐tilted XRD texture measurements and the true out‐of‐plane texture; the weak (311) signal corresponds to an out‐of‐plane <322> texture component. (E) Microstrain and grain size of as‐electroplated ng‐Cu layers in ng/nt‐Cu and ng/cs‐Cu, derived from W–H analysis. (F) High‐resolution TEM image of the ng‐Cu layer in ng/nt‐Cu, with the dotted line marking a GB and arrows indicating stacking faults. (G) Surface EBSD IPF‐z map of the nt‐Cu substrate, with red lines denoting Σ3 twin boundaries and black lines indicating general high‐angle GBs (>10°). (H) Point‐to‐point misorientation and KAM distributions along the scan line in (G), reflecting local orientation gradients. (I) Σ3 twin boundary ratio in nt‐Cu and cs‐Cu substrates.

Similar to the ng/nt‐Cu interface, the ng/cs‐Cu interface also exhibits notable stability: after 7 days of self‐annealing, grain growth is completed across most regions except at the ng/cs interface (Figure S10). However, self‐annealing suppression is significantly more pronounced in ng/nt‐Cu than in ng/cs‐Cu, suggesting an additional stabilizing mechanism. To investigate this, we employed χ‐tilted XRD, which enables selective probing of the ng‐Cu layers while excluding signals from the underlying (111)‐oriented substrates (cs‐Cu and nt‐Cu). The χ‐tilted XRD setup is illustrated in Figure 3A. When χ  =  45°, signals from the underlying (111) Cu substrate, Ti adhesion layer, and silicon are effectively screened (Figure 3B and Figure S24 see for details). For statistical reliability, five samples per group were analyzed (Figure S25).

Analysis of texture coefficients of the atop ng‐Cu layers revealed that the <311> texture coefficient (T_C_(311)) in ng/nt‐Cu is slightly higher and T_C_(111) is slightly lower than in ng/cs‐Cu (both defined at χ  =  45°; Figure 3C). The weak <311> texture defined under χ  =  45° is equal to <322> texture in the out‐of‐film direction, which aligns with TKD IPF mapping results (Figure 3D, Figure S5). Although some studies link thin film stability to crystallographic texture, the weak textures observed here suggest that texture alone cannot explain the stability of the ng‐Cu layer in ng/nt‐Cu.

Microstrain and grain size, extracted using the W–H method (Figure 3E), show that the ng‐Cu layer in ng/cs‐Cu exhibits nearly twice the microstrain of that in ng/nt‐Cu, despite having a larger grain size. This contrast likely reflects the effect of substrate microstructural topography on electroplating nucleation: nt‐Cu substrates, with coarse grains and smooth surfaces, promote uniform nucleation and finer grains (Figure S26A, B), whereas cs‐Cu substrates, with abundant GBs, disrupt nucleation and yield larger grains (Figure S26C, D) [49, 50, 51]. Typically, microstrain increases as grain size decreases [52]; therefore, the lower microstrain of the ng‐Cu layer in ng/nt‐Cu, despite its smaller grains, represents an unusual inverse trend.

STEM and SAED analyses reveal numerous twin boundaries within individual nanograins of the ng‐Cu layer in ng/nt‐Cu (Figure S27A, B). HR‐TEM further shows frequent stacking faults (SFs) intersecting regular GBs across multiple regions (Figure 3F, Figure S27C–F). The twin density of the ng‐Cu layers, estimated from XRD peak asymmetry (see Supporting Information) [53, 54], gives a twin probability (β) of ∼0.48% for ng/nt‐Cu (one twin per 207 (111) planes), compared with ∼0.33% for ng/cs‐Cu (Table S1). Despite inherent uncertainties, the higher β value—corroborated by TEM—confirms a higher twin density in the ng‐Cu layer of ng/nt‐Cu.

Previous experimental and computational studies on face‐centered cubic (fcc) metals demonstrate that the formation of twins and stacking faults from grain boundaries—through partial dislocation emission—can relax GBs and lower the system's energy, especially in metals with low stacking fault energy like Cu [55]. Our observations in the ng‐Cu layer of ng/nt‐Cu align with this: nanoscale twin formation stabilizes GBs and suppresses atomic diffusion along them, particularly near triple junctions [56]. This mechanism also accounts for the anomalously low microstrain of the ng‐Cu layer in ng/nt‐Cu, as GB relaxation and SF formation facilitate atomic rearrangement into a lower‐stress configuration.

Unlike previous reports where twins or SFs are typically formed by deformation or annealing [54,55], our results show that twins are induced by the underlying nt‐Cu substrate during electroplating. Although this phenomenon of substrate‐induced nanotwin formation has been observed in prior studies, its mechanism remains unclear [57]. EBSD analysis of the nt‐Cu surface (Figure 3G) reveals a high density of twin boundaries (red lines). Scanning across these regions yields both “From Point to Point Misorientation” (FPPM) and Kernel Average Misorientation (KAM) maps: FPPM shows a consistent 60° rotation at each twin boundary, while KAM remains unchanged—unlike across regular grain boundaries, where KAM increases (Figure 3H). This distinction arises because KAM analysis excludes crystallographic symmetry.

Based on these results, we reconstructed the atomic surface topography of the nt‐Cu substrate (Figure S28). EBSD further reveals that neighboring regions B and C are related by a 60° rotation (Figure S28B, C), indicating a single Shockley partial dislocation and an incoherent Σ3 twin boundary (Figure S28D). During initial deposition on nt‐Cu, atoms nucleate epitaxially on both regions, inherently introducing a 60° misorientation that transforms into a Σ3 twin boundary as crystals coalesce. The much higher Σ3 boundary density in nt‐Cu compared to cs‐Cu indicates that nt‐Cu substrates enhance Σ3 twin boundary formation during electroplating of the ng layer. The effect diminishes with increasing film thickness, which coincides with the initiation of stress relaxation and self‐annealing processes at the film surface.

### 2.4 Evaluation of Bonding Performance

Our results demonstrate that self‐annealing is effectively suppressed in ng/nt‐Cu, which retains its nanograined microstructure even after 49 days. Here, we highlight the superior post Q‐time bonding performance of ng/nt‐Cu under low‐thermal‐budget conditions. For bonding tests, a long Q‐time of 30 days was selected.

**FIGURE 4:**
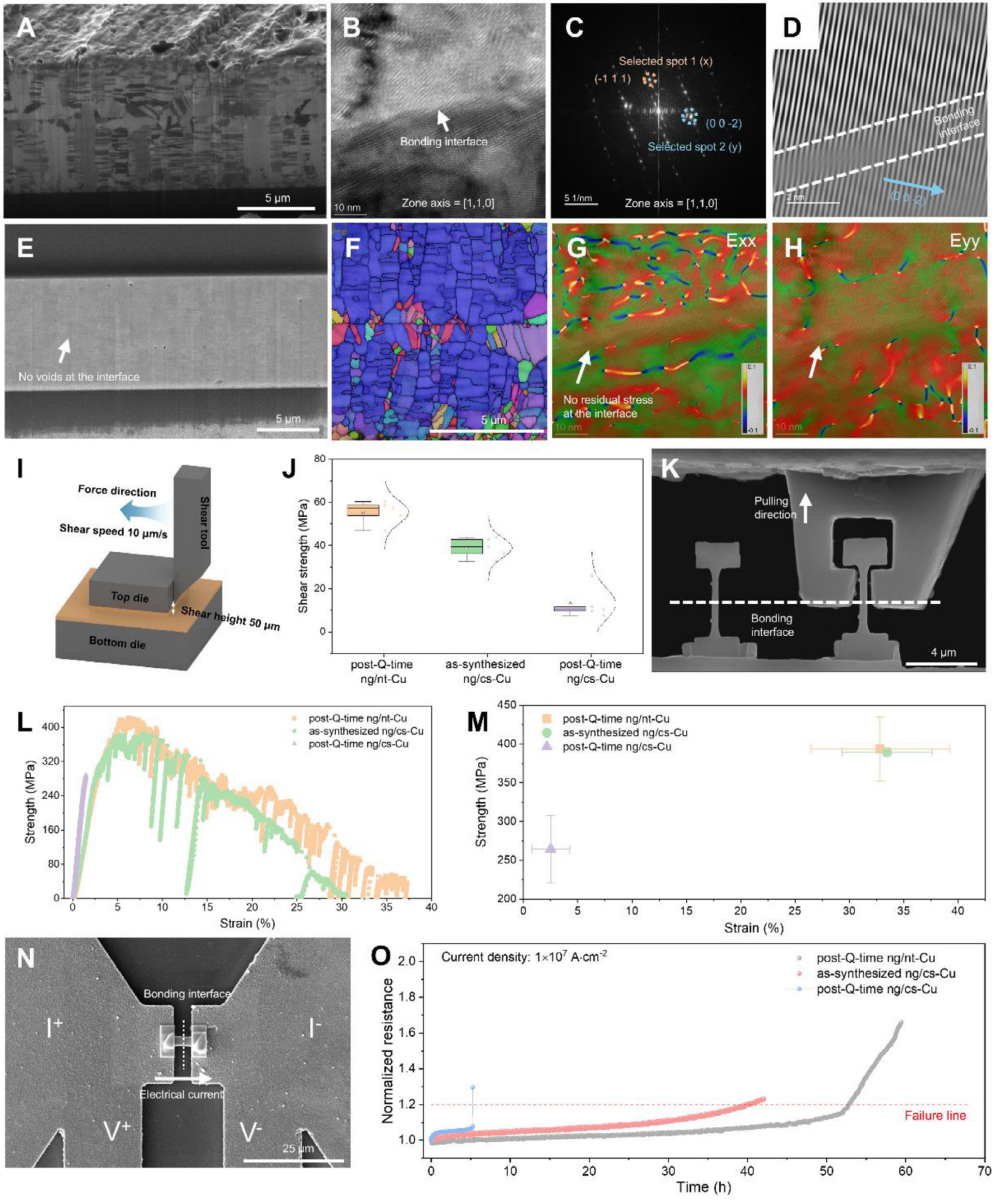
Comprehensive evaluation of post‐Q‐time bonding performance of ng/nt‐Cu. (A) Cross‐sectional FIB image of the bonding interface in post–Q‐time ng/nt‐Cu samples bonded at 200°C for 10 min. (B) High‐resolution TEM image of the bonding region, viewed along the [110] zone axis from the bottom region; the white arrow indicates the bonding interface. (C) SAED pattern obtained via FFT from the bonding interface region in (B). (D) Inverse FFT (iFFT) image from (C) with spot 2 selected; parallel white lines denote the (002̄) lattice planes. (E) Secondary electron SEM image of the bonding interface showing no visible seam (200°C, 10 min); the white arrow marks the interface. (F) EBSD map of the bonding interface under identical bonding conditions. (G, H) Geometric phase analysis (GPA) strain maps showing (G) Exx and (H) Eyy components at the bonding interface corresponding to (D). (I) Schematic of the shear test setup. (J) Shear strength measurements for samples bonded at 200°C for 10 min (five replicates per group). (K) SEM image of the direct‐pull test, with the white dotted line marking the bonding interface and the white arrow indicating the pulling direction. (L) Strain–strength plot from the direct‐pull test under identical bonding conditions. (M) Strength–strain statistics for each sample; each point represents the average of three independent tests, and error bars indicate variation. (N) Electrical testing pattern fabricated via FIB; the segment containing the bonding interface was cut, lifted out, and welded onto a prepared testing substrate. (O) Electromigration test at room temperature; the dashed line marks the 20% resistance increase threshold defined as failure.

Cross‐sectional FIB imaging reveals a well‐bonded interface in post Q‐time ng/nt‐Cu samples bonded at 200°C for 10 min (Figure 4A). The bonded film displays a high density of twins inherent from the pristine nt‐Cu layer. HR‐TEM analysis shows that the bonding interface has transformed into a GB with coherent, parallel crystallographic planes (Figure 4B–D), effectively suppressing electron and phonon scattering—resulting in minimal contact resistance and excellent thermal conductivity [58]. Morphology‐sensitive SEM imaging shows no detectable voids or slits at the bonding interface (Figure 4E), consistent with cross‐sectional optical images (Figure S29). Most of the bonded film exhibits a unidirectional crystallographic structure, with the (111) orientation normal to the bonding interface (Figure 4F). Some nanograins in the middle layer also align with the underlying nt‐Cu, further enhancing coherence. The presence of twin boundaries and strong orientation alignment contributes to enhanced mechanical strength and improved resistance to electromigration [59,60]. Geometric phase analysis strain mapping indicates the absence of residual stress at the interface, supporting long‐term reliability (Figure 4G, H). Robust bonding of post‐Q‐time ng/nt‐Cu is also achieved under other low‐thermal‐budget conditions (300°C for 1 min and 100°C for 60 min; Figure S30).

In contrast, while as‐electroplated ng/cs‐Cu achieves high bonding quality and cross‐interface grain growth at 200°C for 10 min, the post‐Q‐time ng/cs‐Cu sample exhibits a clear unbonded slit under the same conditions (Figure S31), reflecting loss of bonding activity due to self‐annealing during Q‐time. Note that bonding behaviors of as‐electroplated ng/cs‐Cu under various thermal conditions were investigated in our previous work [27]. Interestingly, cross‐interface grain growth is more pronounced in as‐electroplated ng/cs‐Cu than in post–Q‐time ng/nt‐Cu, likely for two reasons: (1) crystallographic defects in the ng‐Cu layer of ng/nt‐Cu are progressively eliminated during the 30‐day Q‐time, particularly within the first three days (Figure 1J), which reduces its bonding activity; and (2) the ng‐Cu layer in ng/cs‐Cu is thicker, providing a larger active volume to support the creep‐like Cu─Cu bonding process. Nevertheless, both bonding interfaces evolve into a grain‐boundary‐like structure with excellent reliability.

To quantify bonding quality, we conducted shear and direct‐pull tests. The shear test setup is shown in Figure 4I. Post–Q‐time ng/nt‐Cu exhibited an average shear strength of 55.4 MPa, significantly higher than the 39.0 MPa of as‐electroplated ng/cs‐Cu (Figure 4J), highlighting the critical influence of microstructure. The high twin boundary density in ng/nt‐Cu impedes dislocation motion, resulting in enhanced strain hardening and ultimate strength (Figure S33) [60]. In contrast, post–Q‐time ng/cs‐Cu showed much lower shear strength (13.0 MPa), indicating inferior bonding.

Direct‐pull tests using dog‐bone‐shaped samples with a central bonding interface (Figure 4K, Figure S34) show that both post–Q‐time ng/nt‐Cu and as‐electroplated ng/cs‐Cu have high tensile strengths (∼394 and ∼389 MPa, respectively; Figure 4L, M, Figure S35, and Movies S1–S3) with plasticity, as evidenced by necking. In contrast, post Q‐time ng/cs‐Cu exhibited lower tensile strength (264 MPa), negligible plasticity, and a brittle fracture mode. This difference in plasticity is attributed to interface quality: only grain boundary‐like interfaces permit dislocation motion and plastic deformation, while defective interfaces cause dislocation pile‐up and brittle failure (Figure S35C) [61]. Importantly, due to the size effect in direct‐pull testing, only the interface—not the bulk microstructure—affects measured strength, explaining the nearly identical strengths for post Q‐time ng/nt‐Cu and as‐electroplated ng/cs‐Cu.

Electromigration tests (Figure 4N, O, Figure S36) further show that post Q‐time ng/cs‐Cu fails quickly, likely due to joint disconnection, whereas post Q‐time ng/nt‐Cu and as‐electroplated ng/cs‐Cu display gradual resistance increases and longer lifetimes, with ng/nt‐Cu exhibiting superior electromigration resistance due to its unique microstructure [59]. Finally, fabrication of ng/nt‐Cu micropad arrays with various dimensions confirms the scalability and industrial feasibility of our method (Figure S37).

## 3 Conclusion

We demonstrate that a bilayer ng/nt‐Cu structure can effectively suppress self‐annealing, preserving its nanograined microstructure throughout extended Q‐time (over 30 days). Our study attributes this exceptional stability to two key factors: (1) the Cu(111) interface, formed by the nt‐Cu underlayer, resists GB migration due to the uniform atomic energy of Cu(111), which limits additional atom adsorption; and (2) the high density of incoherent twin boundaries in the nt‐Cu substrate templates the formation of twins and SFs in the subsequently electroplated ng‐Cu layer. We further demonstrate low‐temperature bonding before and after long Q‐time, with mechanical and electrical evaluation, and fabricate ng/nt‐Cu micropad arrays, confirming the structure's scalability and potential for semiconductor manufacturing. This work provides insight into the fundamental crystallographic mechanisms of electroplated Cu and supports the development of low‐temperature Cu‐Cu bonding techniques, which are particularly relevant for future heterogeneous chip stacking and packaging applications that involve temperature‐sensitive components.

## Funding

Innovation and Technology Commission of Hong Kong, ITF Fund (ITS/104/22); Research Grants Council of Hong Kong, TRS Grant (T46‐705/23‐R); City University of Hong Kong, the startup grant (9380143).

## Conflicts of Interest

The authors declare no conflicts of interest.

## Supporting information




**Supporting File 1**: advs73593‐sup‐0001‐SuppMat.docx.

**Supporting File 2**: advs73593‐sup‐0002‐Movie S1.mp4.

**Supporting File 3**: advs73593‐sup‐0003‐Movie S2.mp4.

**Supporting File 4**: advs73593‐sup‐0004‐Movie S3.mp4.

## Data Availability

The data that support the findings of this study are available from the corresponding author upon reasonable request.
