# Supplementary material for: Interface Engineering Suppresses Self‐Annealing in Electroplated Nanograined Copper for Low‐Temperature Copper‐to‐Copper Bonding
Source: Adv Sci (Weinh). 2026 Jan 4;13(14):e21964. doi: 10.1002/advs.202521964 (PMC12970210; doi:10.1002/advs.202521964)
Supplement: Supplementary file 1 — Supporting File 1: advs73593‐sup‐0001‐SuppMat.docx. [file ADVS-13-e21964-s003.docx]

Supporting Information

Interface Engineering Suppresses Self-annealing in Electroplated Nanograined Copper for Low-temperature Copper-to-Copper Bonding

Gangqiang Peng^1,+^, Xinyi Dong^1,2,+^, Binzhao Li^3,+^, Chuan He^1^, Cong Chen^4^, Licheng Huang^1^, Jian Liu^1^, Yonghan Zhou^1^, Qikai Li^1^, Hao Gong^3^, Yuechen Wang^1,5^, Kaiyu Mu^6^, Sheng-Jye Cherng^6^, Po-Yen Yang^6^, Yang Lu^7^, Yong Yang^3^, Shih-Wei Hung^8,^*, Shien-Ping Feng^1,^*

Experimental Section

Sample preparation

Cu films with distinct microstructures were fabricated by direct current (DC) electroplating from a commercial electrolyte (SS303; Doctech Ltd., Hong Kong, China), comprising 0.48 mol L⁻¹ CuSO₄·5H₂O, 1.90 mol L⁻¹ H₂SO₄, 52 ppm Cl⁻, and multiple proprietary additives. By varying the additive composition, four types of Cu films were obtained: (1) randomly oriented coarse-grained (cg-Cu), (2) (111)-oriented nanotwinned (nt-Cu), (3) high-impurity nanograined (ng), and (4) low-impurity ng Cu. All films were deposited on silicon wafers (total thickness ~625 μm) coated with a 100 nm Ti adhesion layer and a 200 nm Cu seed layer, both sputter-deposited under a working pressure of 13 mTorr using 2000 W (Ti) and 6000 W (Cu) DC power with 132 cm³ STP min⁻¹ Ar flow. The resulting seed layer exhibited a strong out-of-plane (111) texture and is referred to as the cs-Cu substrate. Single-layer nanograined Cu films were deposited directly on the cs-Cu substrate (ng/cs-Cu). For bilayer structures, either cg-Cu or nt-Cu films (~4.41 μm) were first deposited on the cs-Cu substrate at a current density of 4 ASD (40 mA cm⁻²), followed by the deposition of a nanograined Cu layer at 6 ASD for durations of 60 s (~1.32 μm), 80 s (~1.76 μm), 100 s (~2.21 μm), and 120 s (~2.65 μm), respectively, forming ng/cg-Cu or ng/nt-Cu bilayer structures. To ensure comparable surface roughness to the cs-Cu substrate (~1.2 nm), potentiostatic electropolishing at 1.75 V vs. a platinum reference electrode was applied to the cg-Cu and nt-Cu films prior to ng layer deposition. The thicknesses of all electroplated Cu films were measured using a stylus profilometer (Bruker Dektak XT).

Microstructure characterization

Scanning transmission electron microscopy (STEM, Thermo Scientific Talos F200X) was performed at an accelerating voltage of 200 kV using multiple imaging modes, including bright-field (BF) and dark-field (DF), depending on the specific observation needs. An integrated energy-dispersive X-ray spectroscopy (EDS) detector was employed for elemental analysis of impurities. Crystallographic orientation and grain boundary (GB) characterizations were carried out using electron backscatter diffraction (EBSD, Oxford Instruments NordlysNano) and transmission Kikuchi diffraction (TKD), operated on the same platform under different modes. EBSD was conducted at 15 kV, 13 nA with a 15 nm step size, while TKD was performed at 25 kV, 3 nA with the same step size. TKD was utilized for samples with grain sizes below ~100 nm, where EBSD resolution is limited. All orientation maps are presented in the inverse pole figure Z (IPF-Z) direction, corresponding to the out-of-plane orientation of the Cu films. Grain size distribution, band contrast, kernel average misorientation (KAM), and geometrical necessary dislocation (GND) density were extracted from the TKD and EBSD data. Lamella samples for STEM and TKD were prepared using a dual-beam focused ion beam system (FIB, Thermo Scientific Helios 5 CX). A final polishing step at 5 kV and 7 pA was applied to minimize surface damage and remove the amorphous layer induced by ion milling. X-ray diffraction (XRD, Rigaku SmartLab) with Cu Kα radiation in parallel beam geometry was used to determine grain size and microstrain through θ–2θ scans. Operating conditions were 40 kV and 150 mA, with a step size of 0.005° and a scan speed of 5° min⁻¹. Measurements at various χ tilting angles were performed to suppress substrate signals and isolate the response from the ng Cu layer. Further details on χ-tilted XRD are provided in the supplementary text. Atomic force microscopy (AFM, Bruker MultiMode 8) was employed to assess the surface roughness of the samples.

Resistance measurement

The electroplated Cu films were patterned into square shapes with an area of 0.5 cm² (≈ 0.71 × 0.71 cm²) using a custom-designed plating mask. Sheet resistance measurements of the ng layer were conducted using a stage-mounted four-point probe, with the probes positioned at the center of each film. Each data point represents the average of four parallel samples to ensure reproducibility. Details on isolating the resistance contribution of the ng layer from the underlying multilayer conductive structure are provided in the supplementary text.

Impurity analysis

Impurity depth profiling was performed using time-of-flight secondary ion mass spectrometry (ToF-SIMS; IONTOF M6). A 2 keV Cs⁺ ion beam with a current of 63.5 nA was used to sputter a 200 × 200 μm² crater, with a total dose density of 5.45 × 10¹⁸ ions cm⁻². We focused on impurities with detectable and meaningful signals (C, O, S, and Cl). Nitrogen was not included in the TOF-SIMS depth profiling because its signal intensity in Cu is extremely low and close to the detection limit under our measurement conditions. Quantification of C, S, and Cl impurity levels was conducted by referencing standard samples. The microstructural and elemental distribution of impurities, particularly in the form of nanoprecipitates, was analyzed by STEM-EDS. One of the Cl nanoprecipitates is analyzed using a Cs-corrected JEOL JEM-ARM 200F transmission electron microscope operated at 200 keV and equipped with a CEOS imaging aberration corrector. High-resolution high-angle annular dark-field scanning transmission electron microscopy (HAADF-STEM) images were acquired with a convergence semi-angle of 26 mrad and collection semi-angles ranging from 50 to 180 mrad, providing a spatial resolution of up to 0.08 nm. HAADF images were further processed using a DCFI (drift-corrected frame integration) method to enhance image clarity. Strain mapping was performed via geometrical phase analysis (GPA) using the GPA plugin in Digital Micrograph (Gatan). Image drift was corrected using the StackReg (Rigid Body) plugin in ImageJ.

Direct Cu-Cu bonding

Wafers with electroplated Cu films were diced into small (0.5 × 0.5 cm²) and large (1 × 1 cm²) dies. The Cu surfaces were planarized via chemical mechanical polishing (CMP) at 90 rpm for 4 minutes, with a material removal rate of ~40 nm min⁻¹. Following polishing, the dies were sequentially cleaned in an ultrasonic bath with acetone, isopropanol, and an acid solution (37 vol.% HCl:DI water = 1:1 v/v), then rinsed with deionized water and dried under N₂ gas. Prior to bonding, a 20 W N₂ plasma treatment was applied for surface cleaning and activation. Cu–Cu bonding was performed in vacuum (200 Pa) under a compressive stress of 20 MPa, using one of the following bonding conditions: 300 °C for 1 min, 200 °C for 10 min, or 100 °C for 60 min. For post-Q-time bonding studies, samples were stored in vacuum for 30 days prior to bonding.

Bonding quality evaluation

Bonded samples were mounted in conductive epoxy, then ground perpendicular to the bonding plane to expose the interface. Sequential polishing ensued using silicon carbide papers (2000-grit to 4000-grit) followed by 1 μm diamond suspension on a polishing cloth. Optical microscopy (Olympus BX53M) facilitated preliminary interface characterization, whereafter site-specific FIB milling prepared regions for nanoscale analysis. The interfacial microstructure was interrogated using secondary electron SEM to detect topographical defects (e.g., nanovoids and interfacial slits), and FIB-based orientation contrast imaging to resolve crystallographic characteristics. Mechanical assessments comprised: (i) shear testing via an XYZTec bond tester with the shear tool edge precisely aligned to the top-die edge for uniform force application, at a height of 50 μm above the substrate and displacement rate of 10 μm/s (mean strength reported from five specimens per group); and (ii) micro-tensile testing on lamellae lifted from bonded dies using an EasyLift nanomanipulator in an FIB system, mounted onto Mo grids, and sculpted into 400 nm × 400 nm gauge sections centered on the interface. Tensile specimens underwent final FIB polishing at 30 kV/30 pA to mitigate ion-beam artifacts before uniaxial loading under displacement control (Hysitron PI 85 Picoindenter, nominal strain rate 1×10⁻³ s⁻¹), with force-displacement data recorded for stress-strain analysis (three replicates per group). Dynamic tensile deformation (Movie S1–S3) was captured *in situ* via SEM at low electron dose (10 kV, 0.1 nA) to minimize beam effects.

Electromigration test

The patterned wafer for the electromigration test vehicle was first fabricated using standard lithography, Cu electroplating, and etching processes. The test structure follows a typical four-probe configuration (Figure S36). After completing the fabrication, a rectangular micro-stick (with each side measuring 1 μm) containing the bonding interface at its center was prepared from the bonded sample using FIB milling and then mounted onto the test vehicle. Subsequently, an electrical current corresponding to a current density of 1 × 10^7^ A cm⁻² was applied to induce electromigration stress.

Fabrication of ng/nt-Cu micropads arrays

Photolithography was used to define micropatterns for subsequent Cu electroplating. A 6-inch silicon wafer with a Ti adhesion layer and Cu seed layer (as described in the sample preparation section) was used as the substrate. A positive photoresist (PR) was spin-coated onto the wafer at 3000 rpm for 30 s to achieve a uniform thickness of ~10 μm. The coated wafer was then soft-baked on a hot plate at 95 °C for 2 minutes to remove residual solvent and improve PR adhesion. Next, standard UV photolithography was performed using a mask aligner to expose microcircle array patterns in four designated regions on the wafer. Each region had different combinations of pattern pitch (18, 36, 54, and 72 μm) and critical dimension (CD: 10, 20, 30, and 40 μm). The exposure dose and time were optimized according to the PR specifications. After exposure, the wafer was developed in a commercial developer solution for 60–90 s to remove the exposed PR, revealing the desired circular openings. A post-exposure bake at 110 °C for 1 minute was applied to improve pattern definition and dimensional stability. The patterned wafer was rinsed with deionized water and dried with N₂ gas. Electroplating was then carried out through the patterned PR mask to deposit a Cu bilayer. First, a ~4.41 μm thick nt-Cu Cu layer was deposited, followed by a ~1.76 μm ng Cu layer, forming the ng/nt-Cu bilayer Cu micropads. After electroplating, the remaining photoresist was stripped using acetone in an ultrasonic bath, leaving behind the freestanding Cu micropads.

Molecular dynamics

To investigate the atomic-scale grain merging behavior, we constructed simulation domains consisting of a monocrystalline (single-crystal) copper region and a nanograined (polycrystalline) copper region. All structures were generated using the open-source software ATOMSK,[1] based on the face-centered cubic (FCC) lattice of Cu with a lattice constant of 3.615 Å. Five distinct orientations of the single-crystal region—(111), (211), (221), (322), and (331)—were individually modeled to systematically evaluate the orientation dependence of grain merging.

The nanograined region was generated using Voronoi tessellation,[2] containing 40 randomly oriented grains to approximate realistic nanocrystalline structures. The overall simulation domain was a rectangular box approximately 100 × 100 × 100 Å³ in size, with the lower half consisting of the single-crystal region and the upper half composed of the nanograined structure. Periodic boundary conditions were applied along the X and Y directions, while 10 Å vacuum slabs were added at the top and bottom (Z direction) to allow free interface motion, mimicking a semi-infinite slab geometry (Figure S19).

All molecular dynamics (MD) simulations were conducted using the Large-scale Atomic/Molecular Massively Parallel Simulator (LAMMPS) package.[3] Interatomic interactions were modeled using an Embedded Atom Method (EAM) potential,[4] widely used for simulating metallic bonding in copper due to its ability to accurately describe atomic cohesion and local electron density.

Prior to interface formation, the single-crystal region was relaxed under the isothermal-isobaric (NPT) ensemble at 300 K and 0 bar to eliminate residual stress. The nanograined region was then scaled along X and Y to match the in-plane dimensions of the single crystal. The two regions were brought into atomic contact, forming a sharp initial interface. Energy minimization was then performed to eliminate unfavorable high-energy contacts.

To allow atomic rearrangement at the interface, the system was equilibrated in the canonical (NVT) ensemble at 300 K for 200 ps. These equilibrated structures were then used as the initial configuration for subsequent grain merging simulations, also performed under NVT at 300 K for a total duration of 3 ns. The integration timestep was 1 fs. All simulations were repeated with different initial velocity seeds to ensure statistical robustness.

Atomic configurations from all simulation frames were visualized and analyzed using OVITO.[5] The Polyhedral Template Matching (PTM) algorithm was used to identify local atomic structures and monitor the evolution of grain boundary (GB) structures during simulation.[6] PTM compares local atomic configurations to reference crystal templates and provides robust identification even under thermal noise, offering improved reliability compared to the Common Neighbor Analysis (CNA) method.[7]

Atomic potential energy was computed for each atom, and atoms were classified into “crystalline” or “grain boundary” regions based on a threshold of –3.4 eV. This threshold was selected based on reference distributions obtained from pristine single-crystal and nanocrystalline copper (see Supplementary Text). The mean square displacement (MSD) of atoms in the Z direction was also computed to quantify GB motion and interface stability over time.

**Supplementary Text**

Electrical resistance calculation

Since multilayer conductive films can be modeled as parallel circuits,[8] the individual sheet resistance of the ng-Cu layer—subject to time-dependent evolution—can be extracted from the total resistance of multilayer structures by employing an equivalent circuit model (Figure S9). The resistance contributions from the underlying cs-Cu (including Ti adhesion layer), nt-Cu, and cg-Cu layers are constant and time-invariant.

First, the sheet resistance of the bare cs-Cu substrate, $R_{cs}$, is measured. For the ng/cs-Cu, the total measured sheet resistance $R_{tot-1}$ follows:

$$\begin{aligned} \frac{1}{R_{tot-1}}=\frac{1}{R_{cs}}+\frac{1}{R_{ng}}\#(1) \end{aligned}$$

From Eq. (1), the sheet resistance of the ng layer, $R_{ng}$, can be determined.

Similarly, for structures where nt-Cu or cg-Cu layers are electroplated on cs-Cu, the total measured resistances $R_{tot-2}$ and $R_{tot-3}$, respectively, are given by:

$$\begin{aligned} \frac{1}{R_{tot-2}}=\frac{1}{R_{cs}}+\frac{1}{R_{nt}}\#(2) \end{aligned}$$

$$\begin{aligned} \frac{1}{R_{tot-3}}=\frac{1}{R_{cs}}+\frac{1}{R_{cg}}\#(3) \end{aligned}$$

From these equations, the sheet resistances $R_{nt}$ and $R_{cg}$ can be extracted.

For the ng/nt-Cu and ng/cg-Cu bilayer structures, the total resistances $R_{tot-4}$ and $R_{tot-5}$, respectively, are described by:

$$\begin{aligned} \frac{1}{R_{tot-4}}=\frac{1}{R_{cs}}+\frac{1}{R_{nt}}+\frac{1}{R_{ng}}\#(4) \end{aligned}$$

$$\begin{aligned} \frac{1}{R_{tot-5}}=\frac{1}{R_{cs}}+\frac{1}{R_{cg}}+\frac{1}{R_{ng}}\#(5) \end{aligned}$$

Using Eqs. (2)–(5), the time-evolving sheet resistance of the ng layer can be isolated in the ng/nt-Cu and ng/cg-Cu bilayer configurations.

Electroplating impurity characterization

Historically, detecting impurities in electroplated Cu has been challenging due to low concentrations and characterization limitations. Here, aberration-corrected STEM (AC-STEM) analysis of impurity-enriched interface regions in ng/nt-Cu revealed extensive interfacial nanoprecipitates and large intragranular nanotwins (Figure S15). HAADF imaging confirmed the crystallinity of nanoprecipitates, while EDS (Figure S16) identified co-segregation of C and S in nanoclusters, with Cl auto-segregation. ToF-SIMS corroborated the co-mobility of C/H/O/S impurities and the isolated immobilization of Cl. Atomic-resolution AC-STEM (Figure S17) resolved coherent Cl-containing nanoclusters with Z-contrast distinguishing them from the Cu matrix. Geometric phase analysis indicated these nanoclusters locally strain the lattice. According to classical strengthening and Zener pinning theory, such nanoclusters impede both dislocation glide and grain boundary migration.

X-ray diffraction analysis

The transition layer of the (111)-oriented nanotwinned Cu (nt-Cu)—a ~200 nm-thick randomly oriented nanograined (ng) region formed during initial nucleation—must be excluded to avoid undesired XRD signal contributions. To achieve this, we electroplated a 20 μm-thick nt-Cu layer, which exceeds the ~15 μm XRD penetration depth and thereby prevents detection of the transition layer. As shown in Figure S23E, the Si(004) reflection vanishes at a χ tilt angle of 0°, confirming that the nt-Cu layer is sufficiently thick to fully attenuate signals from the underlying Si substrate, Ti adhesion layer, Cu seed, and the transition region.

To isolate diffraction signals solely from the topmost ng layer in both ng/cs-Cu and ng/nt-Cu samples, we leveraged the highly textured nature of their underlying layers. The depth limitation of X-ray is ~15μm. The substrate of ng/cs-Cu consists of single-crystal Si, a Ti adhesion layer, and a (111)-textured Cu seed, while the substrate of ng/nt-Cu comprises only the >20 μm-thick nt-Cu film. By selecting an appropriate χ tilt angle, the diffracted intensity from these single-crystal or strongly textured layers is suppressed, enabling selective detection of the signal originating from the top ng layer.

Figure S23B and C show the XRD measurement configurations at χ = 0° and χ = 45°, respectively. We systematically tested tilt angles of χ = 0°, 15°, 30°, 45°, and 60°, and found that only at χ = 45° are all diffraction signals from the single-crystal Si, Ti adhesion layer, (111)-textured Cu seed, and nt-Cu layer effectively suppressed. This is due to the absence of any crystallographic planes in these highly textured layers that are oriented normal to the XRD diffraction vector at this tilt angle. Therefore, χ = 45° was chosen as the optimal condition to isolate and characterize the diffraction signal from the topmost nanograined layer in both ng/cs-Cu and ng/nt-Cu samples.

The crystallite size and microstrain of our nanograined Cu films were rigorously quantified using the Williamson–Hall (W–H) method,[9] which deconvolutes the Gaussian and Lorentzian components of XRD peak broadening into contributions from finite domain size and lattice distortion, respectively. In this framework, the integral breadth β of each diffraction peak—after subtracting instrumental broadening determined from a LaB₆ standard—is related to the Bragg angle θ by the expression:

$$\begin{aligned} \beta cos\theta=\frac{K\lambda}{D}+4\varepsilon sin\theta\#(6) \end{aligned}$$

where K (≈0.9) is the shape factor for roughly spherical crystallite, λ is the Cu Kα₁ wavelength (1.5406 Å), D is the volume‑weighted average crystallite size, and ε is the root‑mean‑square microstrain. To ensure the highest fidelity in β determination, raw θ–2θ scans for the Cu(111), (200), (220), and (311) reflections were processed in Jade 6 (MDI JADE, Version 6.0, 2013), employing automated background subtraction with a low‑order polynomial, Kα₂ stripping via built‑in profile fitting, and deconvolution of overlapping peaks by pseudo‑Voigt fitting; instrumental profile functions were characterized under identical optics and slit conditions to correct each peak’s FWHM. The resulting β values were plotted as β cos θ versus 4 sin θ, and a weighted least‑squares regression—accounting for uncertainties in β derived from counting statistics—yielded the intercept, Kλ/D, and the slope, 4ε. The exceptionally high linear correlation (R² > 0.9) across four reflections confirms the validity of the isotropic strain and uniform domain shape assumptions inherent to the W–H model.

We estimated the twin probability, β, in the top ng layer on both cs-Cu and nt-Cu substrates via peak‑asymmetry analysis of χ = 45°‑tilted XRD reflections (Cu Kα) acquired on a Rigaku SmartLab diffractometer.[10] Data were collected over 2θ = 40°–95° with a 0.005° step size and a 5°/min scan speed under standard optics, as shown in Figure S24. Peak profiles were first processed in Jade 6—performing background subtraction, Kα₂ stripping, and pseudo‑Voigt deconvolution—to extract accurate β values for each reflection. The asymmetry of the Cu(111) and Cu(200) peaks was quantified by the difference in the positions of the center‐of‐gravity, and the peak maximum:

$$\begin{aligned} \Delta.C.G.\left( 2\theta_{hkl}^{^{\circ}} \right)=(2\theta_{C.G.}^{^{\circ}}-2\theta_{hkl}^{^{\circ}})_{hkl}\#(7) \end{aligned}$$

Where$2\theta_{hkl}^{^{\circ}}$ values correspond to the peak maximum,  $2\theta_{C. G.}^{^{\circ}}$ are the centers of gravity of the point-counted profiles, and $2\theta_{max}^{^{\circ}}$ have been determined from the chart-recorded profiles using the midpoint chord extrapolation method.[11] The twin probability was then calculated as:

$$\begin{aligned} \beta=\frac{\Delta.C.G.\left( 2\theta_{111}^{^{\circ}} \right)-\Delta.C.G.\left( {2\theta}_{200}^{^{\circ}} \right)}{11 tan\theta_{111}+14.6 tan\theta_{200}}\#\left( 8 \right) \end{aligned}$$

The averaged over five independent samples and scans ensures reproducibility. The β value of the ng/nt-Cu is about 0.4825%, i.e., one twin in every 207 (111) planes; while the ng/cs-Cu is about 0.3347%, i.e., one twin in every 299 (111) planes. Such results manifest that twin density in the ng/nt-Cu is notable higher than that in the ng/cs-Cu sample due to the templating effect from the nt-Cu substrate.

Consuming small grains by curvature-driven GB migration

In polycrystalline materials undergoing thermally‑activated grain growth, grains with higher local curvature-i.e., smaller grains-experience a larger capillary driving force toward boundary migration than their larger neighbors. At a curved GB of local curvature κ, the capillary pressure is given by:[12]

$$\begin{aligned} \Delta P=\gamma\kappa\#\left( 9 \right) \end{aligned}$$

where γ is the specific GB energy. Since κ scales inversely with the grain radius (κ ≈ 1/R for a nearly circular two‑dimensional grain), smaller grains (smaller R) possess higher ΔP and therefore migrate more rapidly under the same boundary mobility M (m⁴·J⁻¹·s⁻¹) than larger grains. The normal velocity of GB migration is [12]:

$$\begin{aligned} v_{n}=M\Delta P=M\gamma\kappa=\frac{M\gamma}{R}\#\left( 10 \right) \end{aligned}$$

so that small grains shrink faster (|dR/dt| ∼ 1/R) and are ultimately “consumed” by adjacent, lower‑curvature (larger) grains.

This curvature‑driven coarsening can be formalized by considering the net flux of boundary segments: each GB moves toward its center of curvature, transferring volume from the shrinking grain into its larger neighbors. Integrating the migration law for a two‑dimensional grain yields the parabolic growth law:[13]

$$\begin{aligned} R\left( t \right)^{2}-R_{0}^{2}=2M\gamma t\#\left( 11 \right) \end{aligned}$$

where R_0_ is the initial grain radius. In three dimensions, the boundary velocity depends on the sum of principal curvatures, but the same principle holds: grains of smaller radius exhibit faster recession and vanish first, leading to a statistical shift in the grain‑size distribution toward larger mean sizes.

Real materials often contain additional drag forces —solute atoms, second‑phase particles, or anisotropic boundary energies—that modify this idealized behavior.[14] In the ng/nt-Cu system, the atomically flat and uniformly energetic (111) interface stabilizes regions with high curvature difference by suppressing atom adsorption—and therefore diffusion—along the boundary. This reduced diffusivity in turn inhibits GB migration across the interface. Our demonstration of this canonical interface design offers a powerful strategy for microstructure engineering, not only in advanced alloys but also in other interfacial and surface‐science applications such as oxidation resistance and heterogeneous catalysis.

Molecular dynamics: rationale and analysis of GB structure and dynamics

The approach of combining a single-crystal region with a nanograined region in a controlled atomic model allows us to systematically investigate grain merging phenomena under idealized yet experimentally relevant conditions. The random orientations of the 40 grains in the nanograin region reflect the stochastic nature of grain boundary configurations typically seen in electrodeposited copper films.

Atomic potential energy was used as a reliable metric to distinguish crystalline atoms from those located at grain boundaries. In defect-free single-crystal copper, atoms exhibited potential energies below –3.4 eV. In contrast, atoms in the polycrystalline region showed a broader energy distribution, with a significant number of atoms having values above this threshold due to the structural disorder and incomplete coordination at grain boundaries. Thus, –3.4 eV was used as a conservative threshold to differentiate between ordered and disordered atomic environments. Figure S21 shows the comparative energy distribution in single-crystal and nanocrystalline configurations, justifying this criterion.

The PTM method further enhances the robustness of structural classification by mitigating the influence of thermal vibrations, which can lead to misclassification in methods relying on fixed cutoff distances (e.g., CNA). PTM evaluates the local polyhedral geometry of atoms and maps it to known FCC, BCC, or HCP reference templates, thereby ensuring accurate phase and defect identification even during dynamic evolution.

To quantify the extent of grain boundary motion and structural coalescence, we analyzed atomic displacements along the Z direction, which is orthogonal to the interface. The computed Mean Square Displacement (MSD) allows us to track whether grains remain morphologically stable or undergo boundary migration. Low MSD values indicate limited atomic movement across the interface, implying a stable nanograin morphology. In contrast, elevated MSD values suggest active boundary migration or interface instability, corresponding to grain merging behavior.

Together, these structural and energetic analyses provide a comprehensive and quantitative understanding of the atomic mechanisms governing grain merging, and serve as a strong theoretical complement to our experimental observations.

**Figure S1.** Schematic illustration of the fabrication process of the ng/nt-Cu bilayer.

Figure S2. Cross-sectional STEM analysis of the ng/nt-Cu. (C) Low-magnification STEM bright field image to see the complete structure of the ng/nt-Cu bilayer. Dashed boxes mark regions selected for higher-resolution imaging (A, B, D, E).

**Figure S3. Spatial distribution of GND density in the ng/nt-Cu.** GND density, calculated from orientation gradients in the TKD data set of Figure 1D, is rendered as a colour overlay (blue → low, green → high) on the underlying band-contrast map. The upper ng cap exhibits a dense, interconnected network of GNDs concentrated at grain boundaries, whereas the underlying nt-Cu layer shows markedly lower dislocation content, punctuated only by isolated threading sources.

**Figure S4. TKD analysis of the bottom nt-Cu layer in the ng/nt-Cu. (A)** Cross-sectional IPF-z map of the bilayer; the lower nt-Cu layer is highlighted by the outline and selected for quantitative texture analysis. **(B)** {111}, {200}, and {220} pole figures derived from the indexed TKD data reveal a sharp (111) fiber texture with the <111> axis closely aligned to the film normal. **(C)** Corresponding stereographic triangles confirm the narrow orientation spread (<5° half-width) around the <111> growth direction.

**Figure S5. TKD analysis of the top ng-Cu layer in the ng/nt-Cu. (A)** Cross-sectional IPF-z map of the bilayer; the upper ng layer highlighted by the outline is selected for statistical analysis. **(B)** {111}, {200}, and {220} pole figures reveal a nearly random orientation distribution, in striking contrast to the sharp <111> texture of the underlying nt-Cu Cu. **(C)** Corresponding stereographic triangles further illustrate the weak crystallographic preference of <322>, with maximum orientation densities < 2 times random.

**Figure S6. FIB workflow for preparing cross-sectional TKD/TEM lamellae.** **(A)** Plan-view SEM image of the ng/nt-Cu surface prior to milling. **(B)** Platinum protective strap (~2 µm thick) deposited over the surface of the sample. **(C)** Trench milling on both sides of the strap defines a 10 µm-wide lamella. **(D)** In-situ lift-out and welding of the lamella to a Cu Omniprobe grid. **(E)** Coarse thinning to ~500 nm total thickness. **(F)** Progressive low-kV polishing reduces the central region to ≤100 nm for electron transparency while preserving interfacial detail. **(G)** Final thinning and removal of surface artefacts. **(H)** Completed specimen mounted normal to the grid post for TKD and TEM analyses.

Figure S7. Structural and surface characterization of electropolished substrates. Microstructural and topographic analyses of three substrate types: (A) nt-Cu, (B) cs-Cu, and (C) cg-Cu. (A1–C1) Inverse pole figure (IPF-z) orientation maps acquired via EBSD, revealing distinct crystallographic textures and grain structures. (A2–C2) AFM 2D surface scans, and (A3–C3) corresponding 3D surface reconstructions of the electropolished substrates. Measured surface roughness values (Ra) indicate consistently low nanometric roughness across all substrates: 1.453 nm for nt-Cu, 1.161 nm for cs-Cu, and 1.103 nm for cg-Cu.

**Figure S8. Impurity levels in electroplated Cu films measured by ToF-SIMS.** Time-of-flight secondary-ion mass spectrometry was used to quantify carbon, sulfur, and chlorine—three common additive-derived contaminants—in three electroplated Cu microstructures: ng-Cu, nt-Cu, and cg-Cu films. The ng-Cu plating typically relies on elevated additive concentrations to promote high nucleation density, producing markedly higher impurity uptake: the summed C + S + Cl content reaches ≈283 ppma, versus 62 ppma for nt-Cu and 33 ppma for cg-Cu.

Figure S9. Electrical equivalent-circuit model for multilayer Cu films. (A) Four-point probe arrangement (ammeter A, voltmeter V) used to measure the sheet resistance of a three-layer conductive stack comprising a ng overlayer (Layer 3), an optional intermediate layer—either cg-Cu, nt-Cu, or none (Layer 2)—and a Ti adhesion ∕ cs-Cu bilayer on Si (Layer 1). (B) Parallel-resistor representation of the stack, in which each metallic layer contributes a sheet resistance R_i_; the resistance relationship is therefore: 1/R_tot_=1/R_1_+1/R_2_+1/R_3_. Dashed outlines indicate the configuration when the intermediate layer is absent. See Supplementary Text for details.

Figure S10. Time-resolved FIB cross-section of the ng/cs-Cu. (A) 1 day and (B) 7days after synthesis.

Figure S11. Time-resolved FIB cross-section of the ng/cg-Cu. (A) 1 day, (B) 3 days, and (C) 7 days after synthesis.

Figure S12. Time-resolved FIB cross-section of the ng/nt-Cu. (A) 1 day, (B) 7 days, (C) 14 days, (D) 21 days, (E) 28 days, and (F) 49 days after synthesis.

**Figure S13. Time-dependent resistivity of multilayer Cu films with ng cap of varying thickness.** Normalized sheet resistivity (ρ/ρ_0_, where ρ_0_ is the day-1 value) was tracked for 28 days at room temperature using a four-point probe; symbols give the mean of four specimens and error bars denote ±1 s.d. **(A)** ng/cs-Cu; **(B)** ng/cg-Cu; **(C)** ng/nt-Cu. Four ng-cap thicknesses were prepared by electroplating at 6ASD (A dm⁻²) for 60 s (~1.32 µm), 80 s (~1.76 µm), 100 s (~2.21 µm), and 120 s (~2.65 µm). Across all architectures, thicker ng overlayers accelerates the early-stage resistivity drop associated with microstructural relaxation, with the ng/nt-Cu stack exhibiting the smallest descent degree.

**Figure S14. ToF-SIMS depth profiles of multilayer Cu films.** Left column: ng/cg-Cu; right column: ng/nt-Cu. For each architecture, depth profiles of various elements were acquired (top) in the as-synthesized state, (middle) after 30 days of room-temperature self-annealing, and (bottom) after rapid annealing at 200 °C for 10 min.

**Figure S15. HAADF-STEM of the ng/nt-Cu interface.** **(A1–A3)** and **(B1–B3)** are tilt-series taken from two neighbouring interfacial regions; each triplet was recorded at successive specimen tilts (± ≈ 1°) to enhance diffraction contrast. White dotted circles and arrows highlight nanoscale precipitates that brighten upon tilting, confirming their crystalline nature. Magenta arrows mark abundant {111} twin lamellae within individual nanograins of the overlying ng layer.

**Figure S16. HAADF-STEM imaging and elemental mapping of impurity nanoprecipitates in the ng layer.** **(A–C)** HAADF-STEM micrographs acquired from the same region at three successive specimen tilts (± ≈ 1°). Bright nanoprecipitates (white arrows) exhibit pronounced diffraction-contrast modulation with tilt, confirming their crystalline character. **(D–H)** Corresponding STEM-EDS maps for Cl, C, S, N, and O, respectively.

 **Figure S17. Atomic-resolution STEM and geometric-phase analysis of a Cl-rich** **nanoprecipitate in the ng-Cu layer.** **(A)** Aberration-corrected BF-STEM image showing a ∼5 nm nanoprecipitate embedded in the Cu matrix. **(B)** Drift-corrected frame-integrated (DCFI) HAADF image of the same region; the diagonal line marks the path for the intensity profile in (C). **(C)** Line-profile of integrated HAADF intensities reveals a ∼7 nm-wide low-intensity zone corresponding to the precipitate core. **(D)** Fast-Fourier transform of (B); diffracted spots selected for geometric-phase analysis (GPA) are labelled ① and ②. **(E,F)** In-plane lattice-strain maps Exx and Eyy obtained from GPA (dashed ellipse outlines the precipitate), highlighting tensile strains.

**Figure S18. Atomistic models of various facets. Ball-and-stick representations compare the close-packed Cu(111) surface with higher-index planes: Cu(211), Cu(221), Cu(322), and Cu(331). Yellow spheres denote substrate atoms, while purple spheres highlight atoms in the uppermost layer and step edges. In contrast to the stepped and kinked morphology of high-index surfaces, Cu(111) exhibits a continuous, close-packed surface that is free of monoatomic steps, making it the atomically flattest facet in this series.**

**Figure S19. Initial atomic models for simulations of orientation-dependent grain merging.**
Each atomic model (approximately100 × 100 × 100 Å³; ~10⁶ atoms) consists of a 50 Å-thick, randomly oriented nanograined Cu layer containing 40 Voronoi grains (upper half), joined to a 50 Å-thick single-crystal Cu substrate (lower half). The substrate orientation is (A) (111), (B) (211), (C) (221), (D) (322), and (E) (331). Periodic boundary conditions are applied in the x and y directions, with 10 Å vacuum gaps at the top and bottom to allow interface and free surface evolution along the z direction. Atoms are coloured by polyhedral template matching (PTM): face-centered cubic (F.C.C.) lattice atoms in green, and grain boundary or disordered sites in magenta.

**Figure S20. Grain boundary evolution of ng-Cu on Cu substrates with different orientations.** Time series illustrating grain boundary evolution in a nanograined Cu overlayer (upper half) joined to single-crystal Cu substrate with **(A)** (111), **(B)** (211), **(C)** (221), **(D)** (322), and **(E)** (331) (time stamps indicated above columns). Each panel, from top to bottom, shows: (i) crystal structure coloured by polyhedral-template matching (F.C.C.: green, boundary/defect: magenta); (ii) atomic mean-square displacement (MSD, colour bar 0-8 Å); and (iii) atomic potential energy (colour bar -3.9 eV to -3.5 eV). Dotted boxes indicate the initial location of the single crystal/nanograin interface.

**Figure S21. Atomic potential energy distribution in crystal and grain boundary regions.** Histogram showing the atomic potential energy for a single-crystal Cu substrate (pink) and a nanograined Cu layer (blue). The maximum potential energy in the single crystal is -3.40 eV; therefore, atoms with energies greater than -3.40 eV (indicated by the vertical red dashed line) are classified as grain boundary atoms in all subsequent analyses.

**Figure S22. Atomic mobility on substrates with different orientations.** Mean square displacement (MSD) of atoms as a function of time for each system. Cu(111) exhibits the lowest MSD, indicating exceptional stability, while the higher MSD values for Cu(211), Cu(221), Cu(322), and Cu(331) reflect increased atomic mobility on these high-index facets.

**Figure S23. Surface energy of various Cu surfaces. Slabs of Cu(111) and four high-index facets, Cu(211), Cu(221), Cu(322), and Cu(331), are coloured according to atomic potential energy, ranging from -3.55 eV (blue) to -2.5 eV (red). The Cu(111) surface shows a uniform distribution of low potential energy, whereas the high-index facets reveal alternating regions of high-energy ridge atoms and deeper, lower-energy hollow sites (highlighted with dashed boxes), illustrating the increased energetic instability characteristic of these interfaces.**

**Figure S24. χ-tilted XRD for substrate-signal suppression.** **(A)** Geometry of the χ-tilt experiment: the specimen is held at a fixed tilt angle χ while a θ–2θ scan is acquired. **(B)** Diffractometer set at χ = 0°; **(C)** the same instrument at χ = 45°. **(D)** θ–2θ scans from a cs-Cu substrate and **(E)** from a nt-Cu substrate, each collected at χ = 0°, 15°, 30°, 45° and 60°. Substrate reflections vanish only at χ = 45°, demonstrating that this tilt angle effectively isolates diffraction from the overlying nanograined layer.

**Figure S25. χ = 45°-tilted XRD results.** The θ–2θ scans are from five independent **(A)** ng/cs-Cu and **(B)** ng/nt-Cu specimens.

**Figure S26. Surface EBSD characterization of Cu substrates.** **(A,B)** nt-Cu: IPF map coloured by crystal direction **(A)** and corresponding grain-size histogram (mean ± s.d. = 2.31 ± 0.12 µm) **(B)**. **(C,D)** cs-Cu: IPF map **(C)** and histogram (mean ± s.d. = 110 ± 4 nm) **(D)**. Regular grain boundaries (black) and Σ3 twin boundaries (red) are superimposed in the maps; curves in the histograms are log-normal fits.

**Figure S27. TEM observations of the ng-Cu cap in the ng/nt-Cu.** **(A)** STEM overview revealing nanograins densely intersected by nanotwins. **(B)** Typical SAED pattern of twins. **(C–F)** HR-TEM images of four individual grains (boundaries traced by white dashed lines) showing parallel {111} twin lamellae and frequent Σ3 intersections with grain boundaries, indicative of abundant twin–GB triple junctions.

**Figure S28. Atomic-scale surface reconstruction in the nt-Cu substrate.** **(A)** EBSD IPF map of the film surface (same data set as Figure S25C); Σ3 twin boundaries (red). Asterisks mark two neighbouring grains, B and C, whose orientations differ by a 60° ⟨111⟩ rotation. **(B, C)** Corresponding f.c.c. unit cells visualised along the surface normal, highlighting the twin relationship. **(D)** Atomistic sketch of the resulting incoherent twin boundary: pink line, boundary plane; black arrows, Shockley partials required for the 60°-misorientation relationship; colours distinguish the two mirror lattices.

**Figure S29. Cross-sectional optical micrograph of the Cu–Cu bonding joint.** The post-Q-time ng/nt-Cu bilayer, bonded at 200 °C for 10 min, exhibits a seamless Cu–Cu interface with no discernible voids or gaps, confirming full metallurgical closure.

**Figure S30. Bonding quality of post-Q-time ng/nt-Cu Cu joints under varied thermal budgets.** Each row compares **(A1–C1)** SEM cross-sections for surface/topographical defects, **(A2–C2)** FIB cross-sections for internal microstructure, and **(A3–C3)** EBSD IPF-z maps for crystallographic orientation, after (A) 300 °C, 1 min; (B) 200 °C, 10 min; and (C) 100 °C, 60 min bonding. All conditions yield fully bonded, defect-free interfaces with high orientation continuity across the joint, confirming excellent bonding quality at all tested temperatures and durations.

**Figure S31. Bonding quality of ng/cs-Cu Cu joints: as-synthesized vs. post-Q-time. (A1, A2)** As-synthesized joint after 200 °C, 10 min bonding: SEM and FIB cross-sections show continuous Cu–Cu bonding with pronounced cross-interface grain growth (arrows).
**(B1, B2)** Post-Q-time joint after the same thermal treatment: SEM and FIB reveal an unbonded slit interface with no cross-interface grain merging.

**Figure S32. FIB workflow for cross-sectional STEM specimen preparation at the Cu–Cu bonding interface.** **(A–G)** Sequential SEM images showing FIB milling, lift-out, and thinning steps for preparing a lamella through the bonding interface (white arrows) for subsequent high-resolution STEM analysis.

**Figure S33. Shear strength versus displacement curves for Cu–Cu joints.** Shear test results for post-Q-time ng/nt-Cu, as-synthesized ng/cs-Cu, and post-Q-time ng/cs-Cu joints. Embedded images show representative fracture surfaces for each joint type.

**Figure S34. FIB-based fabrication of dog-bone micro-tensile specimens for bonding strength testing.** **(A–F)** Sequential SEM images illustrate FIB milling steps to isolate, and shape dog-bone microbars containing the Cu–Cu bonding interface (dashed lines in E, F) for micro-tensile testing.

**Figure S35. Micro-tensile testing of Cu–Cu joints bonded at 200 °C for 10 min.** **(A1–C3)** SEM images show micro-tensile bars at different deformation stages: **(A1–A3)** post-Q-time ng/nt-Cu; **(B1–B3)** as-synthesized ng/cs-Cu; **(C1–C3)** post-Q-time ng/cs-Cu. Shrinkage and plastic failure are observed in ng/nt-Cu and as-synthesized ng/cs-Cu, while post-Q-time ng/cs-Cu exhibits brittle, no-plasticity fracture.

**Figure S36. Electrical reliability of Cu–Cu joints stressed at 1 × 10^7^ A cm⁻².** **(A)** Four-probe test vehicle: a FIB-lifted micro-cuboid containing the bonded interface (white box in **B**) is welded into a lithographically defined current/voltage pattern (I⁺/I⁻, V⁺/V⁻). Oxide on the to-be-welded ends on the test vehicle is removed immediately before welding **(C)** to minimise contact resistance. **(D, E)** Completed insertion and weld.

**Figure S37. ng/nt-Cu Cu micropad arrays fabricated at constant areal coverage (24.2 %).
(A)** 52°-tilt SEM of a representative array. **(B)** FIB cross-section of a single pad showing the ng/nt-Cu bilayer structure. Four designs were patterned, each viewed at increasing SEM tilt angles of 0°, 15°, 30°, 45° (columns 1–4): **(C1–C4)** critical diameter (CD) = 10 µm, pitch = 18 µm; **(D1–D4)** CD = 20 µm, pitch = 36 µm; **(E1–E4)** CD = 30 µm, pitch = 54 µm; **(F1–F4)** CD = 40 µm, pitch = 72 µm.

**Table S1.** Twin probability (β) and corresponding stacking-fault spacing in the nanograined layer, derived from χ-tilted XRD peak-asymmetry analysis.

| **Sample** | **Twin probability β (%)** | **Average number of {111} planes per stacking fault*** |
| --- | --- | --- |
| ng/cs-Cu | 0.3347 | 299 |
| ng/nt-Cu | 0.4825 | 207 |

*Calculated as 1 / β (expressed as a decimal), i.e., the mean count of (111) planes separating successive stacking faults.

Movie S1. Real-time micro-tensile test of the post-Q-time ng/nt-Cu Cu joint bonded at 200 °C for 10 min.

Movie S2. Real-time micro-tensile test of the as-synthesized ng/cs-Cu Cu joint bonded at 200 °C for 10 min.

Movie S3. Real-time micro-tensile test of the post-Q-time ng/cs-Cu Cu joint bonded at 200 °C for 10 min.

References

[1]	P. Hirel, *Computer Physics Communications* **2015**, *197*, 212, <https://doi.org/https://doi.org/10.1016/j.cpc.2015.07.012>.

[2]	a) G. Voronoi, *Journal für die reine und angewandte Mathematik (Crelles Journal)* **1908**, *1908* (133), 97; b) Q. Du, V. Faber, M. Gunzburger, *SIAM Review* **1999**, *41* (4), 637, <https://doi.org/10.1137/s0036144599352836>.

[3]	A. P. Thompson, H. M. Aktulga, R. Berger, D. S. Bolintineanu, W. M. Brown, P. S. Crozier, P. J. in 't Veld, A. Kohlmeyer, S. G. Moore, T. D. Nguyen, R. Shan, M. J. Stevens, J. Tranchida, C. Trott, S. J. Plimpton, *Computer Physics Communications* **2022**, *271*, 108171, <https://doi.org/https://doi.org/10.1016/j.cpc.2021.108171>.

[4]	M. S. Daw, M. I. Baskes, *Physical Review B* **1984**, *29* (12), 6443, <https://doi.org/10.1103/PhysRevB.29.6443>.

[5]	A. Stukowski, *Modelling and Simulation in Materials Science and Engineering* **2010**, *18* (1), 015012, <https://doi.org/10.1088/0965-0393/18/1/015012>.

[6]	P. M. Larsen, S. Schmidt, J. Schiøtz, *Modelling and Simulation in Materials Science and Engineering* **2016**, *24* (5), 055007, <https://doi.org/10.1088/0965-0393/24/5/055007>.

[7]	J. D. Honeycutt, H. C. Andersen, *The Journal of Physical Chemistry* **1987**, *91* (19), 4950, <https://doi.org/10.1021/j100303a014>.

[8]	A. I. Khan, A. Ramdas, E. Lindgren, H.-M. Kim, B. Won, X. Wu, K. Saraswat, C.-T. Chen, Y. Suzuki, F. H. da Jornada, *Science* **2025**, *387* (6729), 62.

[9]	a) A. Patterson, *Physical review* **1939**, *56* (10), 978; b) J. Langford, D. Louër, P. Scardi, *Applied Crystallography* **2000**, *33* (3), 964; c) G. Williamson, W. Hall, *Acta metallurgica* **1953**, *1* (1), 22; d) B. Himabindu, N. L. Devi, B. R. Kanth, *Materials Today: Proceedings* **2021**, *47*, 4891.

[10]	K. Vijayan, *J. Mater. Sci. Lett.* **1988**, *7* (9), 993.

[11]	J. Bearden, *Physical Review* **1933**, *43* (2), 92.

[12]	W. W. Mullins, *Journal of Applied Physics* **1956**, *27* (8), 900.

[13]	a) F. J. Humphreys, M. Hatherly, *Recrystallization and related annealing phenomena*, elsevier, **2012**; b) M. Hillert, *Acta metallurgica* **1965**, *13* (3), 227.

[14]	a) J. W. Cahn, J. E. Taylor, *Acta Materialia* **2004**, *52* (16), 4887; b) C. Tweed, B. Ralph, N. Hansen, *Acta metallurgica* **1984**, *32* (9), 1407.
